# Assessment of Changes in Diaphragm Thickness to Predict Extubation Outcomes After Prolonged Upper Abdominal Surgery

**DOI:** 10.7759/cureus.111060

**Published:** 2026-06-17

**Authors:** Soumya S Nath, Samiksha Parashar, Archana Gautam, Poonam Singh, Deepti Sharma, Nandhini Nachimuthu

**Affiliations:** 1 Anesthesiology and Critical Care Medicine, Dr. Ram Manohar Lohia Institute of Medical Sciences, Lucknow, IND; 2 Anesthesiology, Kalyan Singh Super Speciality Cancer Institute, Lucknow, IND

**Keywords:** diaphragmatic thickness fraction, diaphragmatic ultrasound, extubation failure, mechanical ventilation, postoperative pulmonary complications, ventilator weaning

## Abstract

Background: Prolonged upper abdominal surgery causes postoperative pulmonary complications, such as weaning failure. The diaphragmatic thickening fraction (DTF) is regarded as the surrogate of the breathing workload and correlates well with diaphragm strength. Compromised diaphragm function negatively influences ventilation, leading to difficulty in weaning from a mechanical ventilator. The objectives of the study were to estimate the changes in diaphragm thickness and the best cutoff value that will predict extubation failure at the end of prolonged upper abdominal elective surgery.

Methods: In this prospective observational study, 120 patients (aged 18-70 years) of either sex and with American Society of Anesthesiologists physical status I or II, scheduled to undergo upper abdominal surgery lasting four hours or more, were included. Patients taking more than 30 minutes to extubate from the time of administration of the reversal agent were categorized as failure to extubate. The DTF was assessed via ultrasonography.

Results: Twenty-five (20.83%) patients had failed extubation. Sex (*P* = 0.001) and pre-extubation DTF (*P* < 0.001) were identified as significant variables distinguishing patients who were successfully extubated from those who failed extubation. Multivariate logistic analysis showed that female sex (odds ratio (OR) = 0.233; *P *= 0.010) and pre-extubation DTF (OR = 1.071; *P *= 0.001) were significantly associated with successful extubation. A pre-extubation DTF cutoff of >22.63% predicted successful extubation with a sensitivity of 88.04%, a specificity of 56.0%, and an area under the receiver operating characteristic curve (AUC-ROC) of 0.752

Conclusions: Diaphragmatic dysfunction is common after prolonged upper abdominal surgery, and DTF may help identify patients at risk of extubation failure.

## Introduction

Prolonged upper abdominal surgery causes postoperative pulmonary complications (PPCs) like alveolar hypoventilation, a decrease in functional residual capacity, atelectasis, and diaphragmatic dysfunction (DD) [1,2]. Being the primary muscle of inhalation (contributing 60%-80% of the ventilation), compromised diaphragm function negatively influences the ventilation, resulting in impaired gas exchange [3]. Continued DD may increase the probability of pneumonia and re-intubation in the postoperative period. DD, as assessed by decreased diaphragmatic excursion on ultrasonography, results in a shift from abdominal to predominantly rib cage breathing. This shift, in turn, causes postoperative atelectasis and hypoxemia in patients with upper abdominal surgeries [4].

It is reported that diaphragm thickness assessment reflects muscle strength, and patients with DD are difficult to wean from the ventilator. Animal and human studies have shown that patients develop diaphragm atrophy to varying degrees within 12-24 hours of mechanical ventilation, which worsens over time, and that half develop sustained diaphragm weakness beyond 24 hours [2]. In experimental studies, DD has been observed to occur within two hours of mechanical ventilation [5].

Diaphragmatic ultrasonography has been studied in patients undergoing cardiac surgery to predict successful weaning from the ventilator or to predict PPCs [6,7]. Diaphragmatic thickening fraction (DTF) has been proposed as a surrogate criterion to estimate the workload of breathing, and literature reports that it correlates well with diaphragm strength [6-8].

We hypothesized that prolonged elective upper abdominal surgery could lead to DD, thereby influencing extubation outcomes. This study aims to assess changes in DTF after prolonged elective upper abdominal surgery and to evaluate their effect on extubation outcomes. The primary objective was to determine the incidence of DD following prolonged upper abdominal surgery. The secondary objectives were to evaluate the association between diaphragmatic thickness variables and extubation outcomes and to determine the optimal DTF cutoff value for predicting extubation outcomes at the end of prolonged upper abdominal surgery.

## Materials and methods

**Figure 1:**
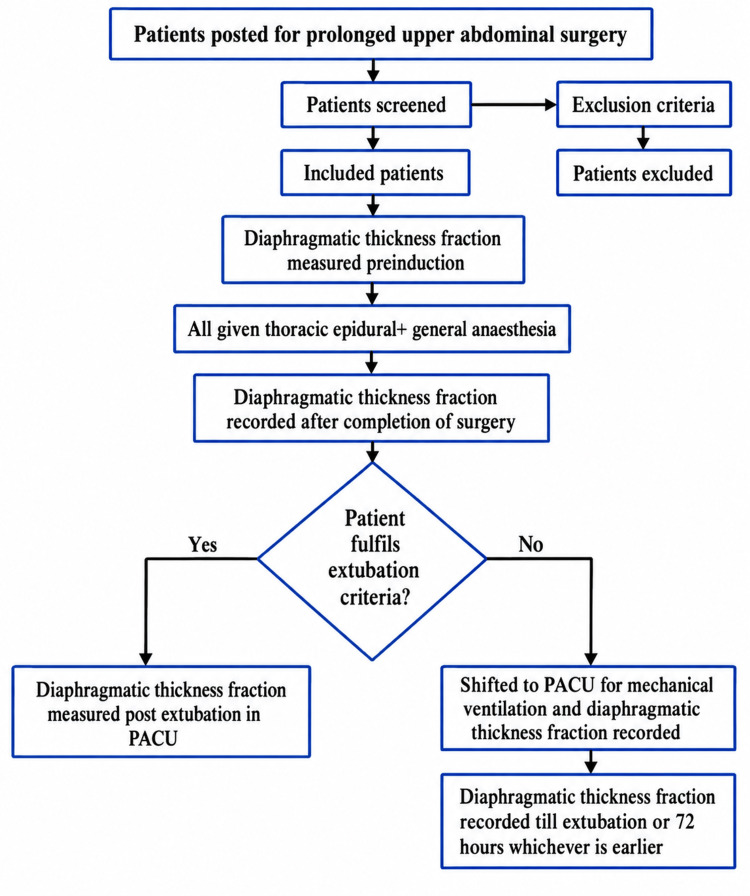
Workflow of the study as per STROBE guidelines. Image credit: Soumya Sankar Nath. STROBE, Strengthening the Reporting of Observational Studies in Epidemiology; PACU, post-anesthesia care unit

The Strengthening the Reporting of Observational Studies in Epidemiology (STROBE) guidelines were followed in this single-center, prospective, observational cohort study [9,10]. The study was conducted in the Department of Anesthesiology at a tertiary care center from April 15, 2023, to August 15, 2024. The study protocol was approved by the Institutional Ethics Committee (IEC No. 172/22) and registered with the Clinical Trials Registry-India (CTRI/2023/04/051308; registered on April 5, 2023). Patient recruitment commenced on April 15, 2023. Written informed consent was obtained from all participants for study participation and the use of their data for research and educational purposes. The study was conducted in accordance with the principles of the 2013 Declaration of Helsinki and Good Clinical Practice guidelines (Figure 1).

Consenting adult patients (18-70 years) of either sex (sex of participants was defined based on self-report) and with American Society of Anesthesiologists physical status I or II who were planned to undergo elective upper abdominal surgery under general anesthesia with a thoracic epidural, and with an expected surgery duration of four hours or more, were screened for eligibility. Exclusion criteria included patients with a history of intensive care unit admission, those already on inotropic support, patients with COPD, and individuals with any disease causing respiratory muscle weakness, such as Guillain-Barré syndrome, cervical spine injury, or electrolyte abnormalities. These patients were excluded because of unclear feasibility and a high susceptibility to delayed extubation through multiple mechanisms.

General anesthesia and a thoracic epidural were administered as per institutional protocol to every patient. Anesthesia was maintained with 1-1.5% isoflurane, supplemented with boluses of vecuronium and fentanyl. The bispectral index was maintained between 40 and 60. Intermittent positive-pressure ventilation was initiated with oxygen and air (40:60) at a tidal volume of 6-8 mL/kg, a respiratory rate of 12-14 breaths per minute, and a positive end-expiratory pressure (PEEP) of 5 cm H₂O. End-tidal carbon dioxide was maintained between 35 and 45 mmHg. At least one hour before the end of surgery, a 0.2% ropivacaine infusion at 4-6 mL/hour was started via the epidural route to ensure that the patient was pain-free after surgery. At the end of surgery, when the train-of-four ratio was greater than 0.9, the neuromuscular block was reversed with intravenous neostigmine 0.04 mg/kg and intravenous glycopyrrolate 0.005 mg/kg. Patients were extubated when they fulfilled the predefined objective criteria for extubation (hemodynamically stable, minimal or no inotropic support, awake, able to protrude the tongue on command, able to sustain a head lift for more than 10 s, core body temperature of at least 36.5 °C, normal partial pressure of arterial carbon dioxide, PaO₂/FiO₂ ratio greater than 250, spontaneous tidal volume greater than 5-6 mL/kg, and maximum inspiratory pressure of -20 cm H₂O).

Patients taking more than 30 minutes to extubate from the time of reversal agent administration were categorized as having delayed extubation. Patients with failed extubation were followed up for the time to extubation.

Ultrasound of the diaphragm was performed on the right side using a linear probe (7-12 MHz) (Sonosite S II, Fujifilm Sonosite Inc., Bothell, WA) via an apposition-zone approach between the 8th and 10th intercostal spaces. All ultrasound examinations were performed by a single operator trained in lung ultrasound with more than five years of experience to avoid inter-operator variability. All measurements were obtained with the patient in a 20°-40° propped-up position. Ultrasound measurements were recorded and averaged over three respiratory cycles. Diaphragm thickness was assessed at the end of inspiration (TEI) and at the end of expiration (TEE). DTF was calculated as (TEI - TEE)/TEE × 100 and expressed as a percentage.

TEI, TEE, and DTF were recorded at three time points: baseline (5 minutes after giving an injection of midazolam, 0.02 mg/kg, in the preoperative room), pre-extubation (5 minutes after administration of the reversal agent), and after extubation (30 minutes after extubation).

All measurements were performed and recorded by the same investigator. Covariates that were recorded were selected based on their clinical relevance and reported association with perioperative extubation outcomes in the previous literature. Potential confounders included demographic parameters (age, sex, height, weight, and body mass index), serum biochemical markers (hemoglobin, serum bilirubin, serum albumin, serum sodium, and serum creatinine), and intraoperative data (inotrope requirement, pre-extubation hemoglobin, and pre-extubation serum sodium).

The sample size was calculated based on a previous study, where *P* represents the estimated proportion of delayed extubation in the population. Assuming a 30% prevalence of diaphragm dysfunction, a 92% confidence level, and a precision of ±8%, the required sample size was estimated to be 100. Considering a dropout rate of 10%, we planned to enroll 120 patients in this study [5].

The data collected from these patients were subjected to statistical analysis. The normality of the data distribution was assessed using the Kolmogorov-Smirnov test. Continuous variables were described using the mean ± standard deviation or the median with interquartile range (IQR). Categorical variables were summarized as frequencies and percentages. Differences between groups were compared using the chi-square test or Fisher’s exact test for categorical data and Student’s unpaired t-test or the Mann-Whitney U test for continuous data, as appropriate. Collinearity was assessed among the significant variables. Binary logistic regression was used to examine the association between extubation outcome and significant covariates. Results are presented as odds ratios (ORs) and standard errors. The discriminatory value of pre-extubation DTF in predicting successful extubation was assessed using receiver operating characteristic (ROC) curve analysis. The optimal cutoff value for dichotomizing the population was calculated using Youden's method. All comparisons were two-tailed, and *P* < 0.05 was considered statistically significant.. Statistical analyses were performed using SPSS 21.0 (IBM Corp., Armonk, NY) and Python 3.13.2 (Python Software Foundation (PSF), Beaverton, OR).

## Results

A total of 137 patients were screened for eligibility, and 120 patients were included based on the inclusion and exclusion criteria. Among them, 95 (79.17%) patients were successfully extubated at the end of surgery, while 25 (20.83%) patients could not be extubated and required postoperative mechanical ventilation. No patient required reintubation. No patients were lost to follow-up, and no data were missing for the primary or secondary outcomes. All patients who failed extubation were subsequently extubated after a mean duration of 240 ± 46.0 minutes. The mean ± SD age of all included patients was 48.59 ± 14.16 years. Table 1 shows the comparison of patient characteristics, laboratory data, and diaphragm variables. Compared with patients who failed extubation, those who were successfully extubated had significantly higher pre-extubation DTF. Carcinoma of the gallbladder (34, 28.3%) was the most common diagnosis among the included patients. Table 2 presents the distribution of diagnoses among the included patients.

**Table 1:** Comparison of different variables between the two groups. The data are presented as median (IQR) or *n* (%). Continuous variables were compared using Student's unpaired t-test or the Mann-Whitney U test, and categorical variables were compared using the chi-square test. A *P*-value < 0.05 was considered statistically significant. *Significant. TEI, diaphragmatic thickness at end inspiration; TEE, diaphragmatic thickness at end expiration; DTF, diaphragmatic thickening fraction; BMI, body mass index; Hb, hemoglobin; Sr, serum

Variable	Extubated (*N* = 95)	Not extubated (*N* = 25)	U statistic	*Z*/*X*^2^	*P*-value
Age (years)	48 (36-61)	52 (43-66)	1061.0	-1.911	0.056
Sex, *n* (%)					
Male	43 (45.3)	20 (80)	-	9.577	0.001*
Female	52 (54.7)	5 (20)			
Site of incision					
Midline	68 (71.6)	17 (68)	-	0.1227	0.726
Subcostal	27 (28.4)	8 (32)			
Height (cm)	164 (158-168)	164 (158-170)	1309.5	-0.422	0.673
Weight (kg)	60 (54-68)	62 (56-68)	1251.0	-0.772	0.440
BMI (kg/m^2^)	22.67 ± 3.17	22.81 ± 3.11	1352.5	-0.162	0.825
Baseline Hb (g/dL)	12.4 (11.8-13.8)	12.4 (11.3-13.9)	1303.5	-0.456	0.648
Sr bilirubin (mg/dL)	0.8 (0.5-2.8)	2.6 (0.34-6.6)	1331.5	-0.288	0.773
Sr albumin (g/dL)	3.8 (3.6-4.2)	3.8 (3.2-4.3)	1254.0	-0.759	0.448
Sr creatinine (mg/dL)	0.63 (0.41-0.81)	0.62 (0.42-0.9)	1373.5	-0.036	0.971
Baseline Sr sodium (mEq/dL)	140 (138-142)	140 (138-142)	1120.0	-1.581	0.114
Pre-extubation hemoglobin (g/dL)	10.8 (9.8-11.8)	10.3 (8.8-11.6)	1149.0	-1.384	.167
Pre-extubation Sr sodium (mEq/dL)	138 (136-140)	136 (134-138)	1092.0	-1.756	.079
Need for inotrope/vasopressor					
Yes	5 (5.3)	1 (4)	-	0.067	0.797
No	90 (94.7)	24 (96)			
Baseline TEI (mm)	8.5 (7.6-9.2)	7.3 (6.5-9.7)	961.0	-2.510	0.12
Baseline TEE (mm)	6.2 (5.7-6.8)	5.2 (4.5-7.3)	1062.5	-1.901	0.057
Baseline DTF (%)	36.1 (29.7-44.3)	31.3 (24.6-46.2)	1191.0	-1.130	.258
Pre-extubation TEI (mm)	5.5 (4.8-5.7)	5.3 (4.4-6.3)	1294.0	-0.513	.608
Pre-extubation TEE (mm)	3.9 (3.2-4.3)	4.3 (3.6-5.2)	980.5	-2.392	.017*
Pre-extubation DTF (%)	40.8 (31.4-51.7)	21.6 (16.2-29.5)	361.5	-6.103	<0.001*

**Table 2:** Distribution of diagnoses among the included patients. CBD, common bile duct

Diagnosis	*n* (%)
Carcinoma gallbladder	34 (28.3)
Periampullary cancer	25 (20.8)
Carcinoma colon/cecum/rectum	24 (20)
Choledochal cyst	11 (9.2)
Carcinoma stomach	8 (6.7)
CBD stone	8 (6.7)
Biliary stricture	4 (3.3)
Miscellaneous	6 (5)

We found that the incidence of delayed extubation in patients following prolonged upper abdominal surgery was 25 (20.83%). DTF was evaluated and compared at baseline and pre-extubation (Figure 1). We observed a significant increase in pre-extubation DTF compared to baseline in patients who were successfully extubated (*P* < 0.001). In contrast, a significant decline in pre-extubation DTF from baseline was noted in patients who failed extubation.

The variables found to be significant (sex, preextubation TEE, and pre-extubation DTF) were assessed for collinearity. A strong negative correlation was observed between pre-extubation TEE and pre-extubation DTF (*r* = -0.770; *P* < 0.001). As they conveyed similar information, the pre-extubation TEE variable was excluded from further analysis. Multivariate logistic regression revealed that female sex and pre-extubation DTF were significantly associated with successful extubation (Table 3).

**Table 3:** Multivariate logistic regression analysis of the association between different variables and extubation success. *Significant. A *P*-value < 0.05 is considered significant. OR, odds ratio; DTF, diaphragmatic thickening fraction

Variable	Coefficient (*B*)	Exp(*B*) OR	Standard error	*P*-value
Sex (0 = Female, 1 = Male)	-1.456	0.233	0.567	0.010*
Pre-extubation DTF	0.069	1.071	0.021	0.001*
Constant	-0.070	0.932	0.804	0.932

**Figure 2:**
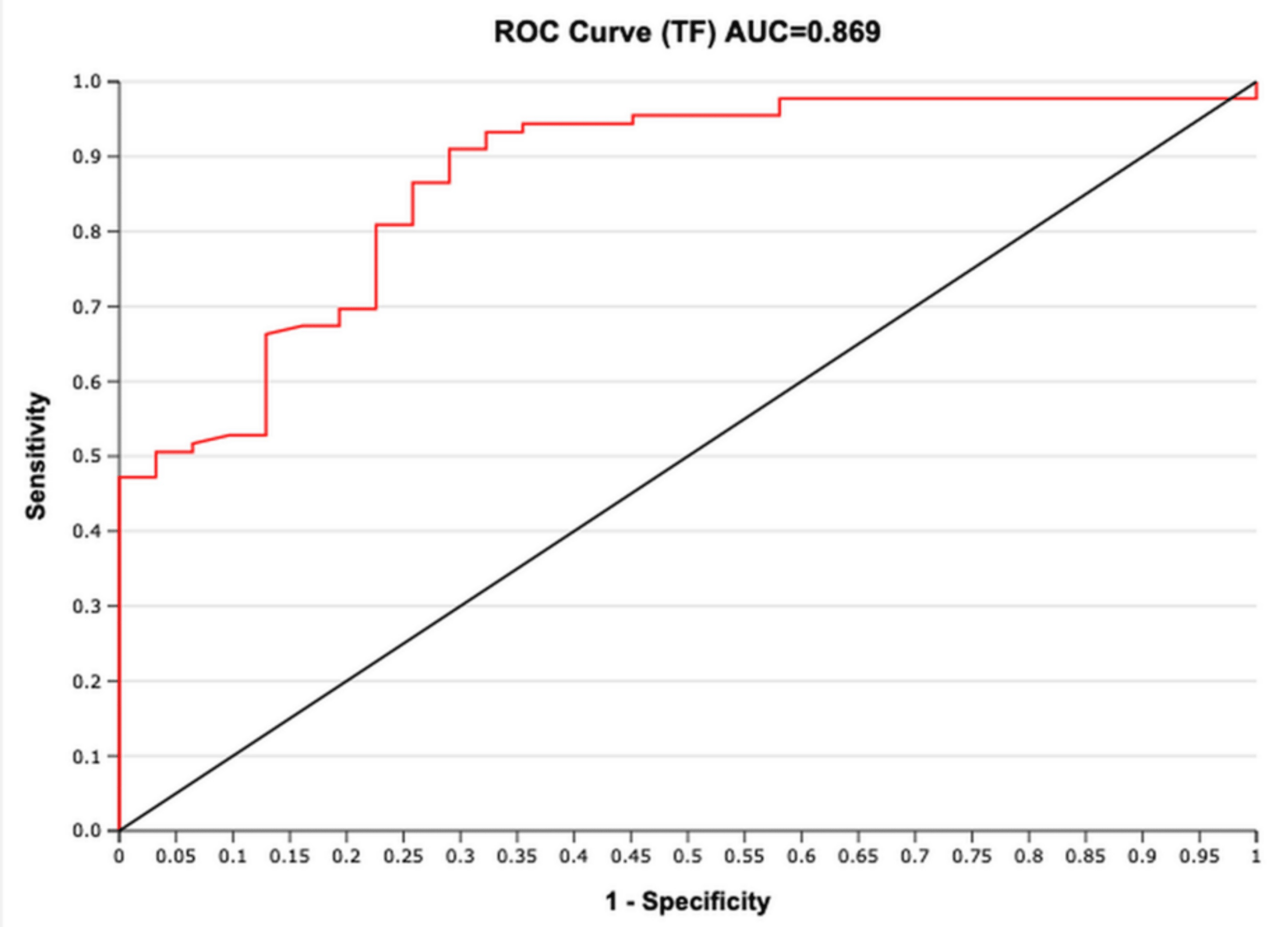
Receiver operating characteristic (ROC) curve to determine the optimal cutoff value of preextubation DTF for predicting successful extubation after prolonged upper abdominal surgery. Trend of DTF measurements at baseline and pre-extubation. Values are presented as median values. Continuous variables were compared using the Mann-Whitney U test. DTF, diaphragmatic thickening fraction

We plotted an ROC curve to determine the optimal cutoff value of pre-extubation DTF for predicting successful extubation after prolonged upper abdominal surgery (Figure 2). We found that a pre-extubation DTF of more than 22.63% predicted successful extubation with a sensitivity of 88.04% and a specificity of 56.0% (ROC AUC = 0.752).

## Discussion

Our study is the first to evaluate diaphragm variables as predictors of extubation outcome after prolonged upper abdominal surgery. A total of 25 (20.83%) patients included in our study failed extubation. The highlight of this study was the significant independent association between pre-extubation DTF and extubation outcome. We observed that a pre-extubation DTF of more than 22.63% predicted successful extubation with a sensitivity of 88.42% and a specificity of 56.0%.

In our study, 83 (69.17%) of the included patients had a diagnosis of carcinoma of the gallbladder, periampullary carcinoma, or cancer of the colon, rectum, or cecum, all of which have a mean age at diagnosis of around 51, 47, and 47 years, respectively [11-13]. This corresponds to the mean age of the patient population in our study (48.59 ± 14.16 years). We found no statistically significant differences in age, height, weight, or BMI between patients who were successfully extubated and those who experienced delayed extubation.

Extubating patients after surgery is always preferred, but some patients fail to fulfill the criteria for successful extubation and require postoperative mechanical ventilation. Identifying patients at risk of delayed extubation could improve clinical decision-making.

We know that the diaphragm is the primary inspiratory muscle, and its impaired function has been linked to prolonged mechanical ventilation [14,15]. DD has been reported as a common occurrence in the intensive care unit and after cardiac or thoracic surgery. It has been shown to delay ventilator weaning in such patients [16,17]. The incidence of DD has not been evaluated in other perioperative settings, and ours is the first study to report the incidence of DD in patients undergoing upper abdominal surgery. We found that the incidence of delayed extubation in patients following prolonged upper abdominal surgery was 25 (20.83%). A similar incidence of DD has been reported among patients undergoing cardiovascular surgery, recipients after bilateral lung transplant, and patients who underwent esophageal cancer resection surgery [18-20].

During active breathing, thickening of the diaphragm has been reported to reflect the magnitude of diaphragmatic effort [1]. Also, DTF has been reported to be a good predictor of weaning outcomes [14]. Low diaphragm thickening is defined as a DTF of less than 20% [6,16]. A DTF below 30% was considered a marker of possible delayed extubation [1]. In our study, we evaluated DTF. We found that patients who failed extubation had a significantly lower pre-extubation DTF than those who succeeded, while patients who succeeded had a significantly higher pre-extubation DTF. Thus, patients developing DD, as assessed by pre-extubation DTF after prolonged upper abdominal surgery, seemed to be at a higher risk of failed extubation. We did not follow the patients until DTF returned to preoperative levels. However, a study by Tralhão et al. monitored postoperative patients until the fifth postoperative day (POD) and observed the return of DTF to preoperative levels by the fifth POD [20].

Independent predictors of extubation outcome identified in our study were sex and pre-extubation DTF. We found that each unit increase in pre-extubation DTF increased the odds of successful extubation by 1.07. In concordance with our findings, DTF, being a direct measure of diaphragmatic contractility and strength, has been reported to predict extubation outcome in a meta-analysis of 14 studies (OR 4.80, 95% confidence interval (CI) 3.86-5.97, sensitivity 81.48%, and specificity 86.86%) [21].

We also observed that sex is an independent predictor of extubation outcome, with males having lower odds of successful extubation than females. The association between sex and extubation outcome has been reported in the literature, with conflicting results. Thille et al. performed a post hoc analysis of data from a multicenter clinical trial involving ICU patients deemed at increased risk of delayed extubation and found that males had a higher reintubation rate within 48 hours than females (11.0% vs. 6.0%; *P* = 0.038). In their analysis, after adjusting for other variables, male sex remained independently associated with an increased risk of reintubation within seven days [22]. A study of 6583 ICU patients scheduled for extubation found that the delayed extubation rate was higher in females than in males (7.7% vs. 5.3%; *P* < 0.001) [23]. Another study of 218 ICU patients found no difference in the incidence of delayed extubation between females and males (11/82 vs. 23/136) [24]. These differences may be attributed to variations in study design and patient populations. Additionally, comorbidities and hormonal factors may have contributed to discrepancies in extubation outcomes between the two sexes [25].

We examined pre-extubation DTF and plotted an ROC curve to determine the optimal cutoff value of pre-extubation DTF for predicting extubation outcome after prolonged surgery. We found that a pre-extubation DTF of more than 22.63% predicted successful extubation with a sensitivity of 87.4% and a specificity of 56.0% (AUC-ROC = 0.752, 95% CI 0.640-0.864; *P* < 0.001) (Figure 2). Our findings are supported by a similar study by Boscolo et al., who analyzed DTF in 44 patients who underwent bilateral lung transplant and reported that the cutoff value that best predicted successful weaning was 29% (AUC 0.88, 95% CI 0.73-0.99) [18].

Our study had certain limitations. First, diaphragm thickness was assessed on the right side only due to easier access and a better acoustic window than on the left, where a gastric gas shadow may obscure the diaphragm. Second, this was a single-center study with a small sample size, and the study population was restricted to patients undergoing elective surgeries. Third, as we excluded patients with COPD, it is questionable whether the findings can be extrapolated to patients with intrinsic PEEP. Fourth, there were relatively few delayed extubation events, and the proposed DTF cutoff showed moderate predictive performance. Finally, we did not assess longer-term postoperative outcomes such as reintubation, postoperative pulmonary complications, ICU length of stay, and hospital length of stay.

## Conclusions

We conclude that DD is common after prolonged upper abdominal surgery. Independent predictors of extubation outcome found in our study were sex and pre-extubation DTF. We found that each unit increase in pre-extubation DTF increased the odds of successful extubation by 1.07. Pre-extubation DTF is an independent predictor of extubation outcome. A DTF >22.63% predicts the chances of successful extubation after prolonged upper abdominal surgery. A change in DTF could be an important adjunct to standard extubation readiness assessment. Thus, although pre-extubation DTF may help identify patients at risk of delayed extubation after prolonged upper abdominal surgery, larger multicenter studies with standardized extubation criteria and external validation are needed.
